# Engineered Collagen Matrices

**DOI:** 10.3390/bioengineering7040163

**Published:** 2020-12-16

**Authors:** Vaidehi A. Patil, Kristyn S. Masters

**Affiliations:** Department of Biomedical Engineering, University of Wisconsin-Madison, Madison, WI 53705, USA; vpatil4@wisc.edu

**Keywords:** collagen, extracellular matrix, scaffolds, tissue engineering

## Abstract

Collagen is the most abundant protein in mammals, accounting for approximately one-third of the total protein in the human body. Thus, it is a logical choice for the creation of biomimetic environments, and there is a long history of using collagen matrices for various tissue engineering applications. However, from a biomaterial perspective, the use of collagen-only scaffolds is associated with many challenges. Namely, the mechanical properties of collagen matrices can be difficult to tune across a wide range of values, and collagen itself is not highly amenable to direct chemical modification without affecting its architecture or bioactivity. Thus, many approaches have been pursued to design scaffold environments that display critical features of collagen but enable improved tunability of physical and biological characteristics. This paper provides a brief overview of approaches that have been employed to create such engineered collagen matrices. Specifically, these approaches include blending of collagen with other natural or synthetic polymers, chemical modifications of denatured collagen, de novo creation of collagen-mimetic chains, and reductionist methods to incorporate collagen moieties into other materials. These advancements in the creation of tunable, engineered collagen matrices will continue to enable the interrogation of novel and increasingly complex biological questions.

## 1. Introduction

Collagen is ubiquitous throughout the animal kingdom and is the most prevalent protein in mammals. Evolutionarily, collagen enabled the transition from single-celled organisms to multi-cellular animals [1], and it is now recognized as an indispensable family of molecules with versatile roles. Collagens constitutes one-third of all protein present in humans and are found within the extracellular matrix (ECM) [2]. This superfamily of proteins consists of at least 46 types of polypeptide chains that make up the 28 types of collagen molecules discovered thus far in vertebrates [3,4]. As collagen is so prevalent within the body, it is found in tissues such as skin, bone, ligaments, tendons, cartilage and the cornea [5].

To meet the needs of these diverse and specialized tissues, the different collagen types demonstrate varying structure, morphology and function. However, despite this diversity, all collagens exhibit a unifying feature that classifies molecules as belonging to the collagen family, namely a repeating Gly-X-Y motif (wherein X and Y may be substituted with any amino acid, but most frequently with proline and 4-hydroxyproline, respectively [6]). This repeated Gly-X-Y sequence allows the assembly of three left-handed polypeptide strands into a right-handed triple helical molecule, thereby forming the characteristic structural feature of collagens [7,8]. 

The assembly of collagen is an entropy-driven process wherein collagen sub-units assemble themselves into fibrils, albeit with tissue-specific variations. Fibroblasts play important roles in manufacturing the monomeric subunits that form collagen, with the secondary structure dependent on the amino acid sequence of the protein, along with essential post-translational modifications such as hydroxylation and glycosylation. After these modifications, the pro-α chains self-assemble into a triple-helical procollagen molecule that is secreted by the fibroblasts to the extracellular environment, and pro-peptide extension pieces cleaved to yield collagen molecules [9,10,11]. 

The diversity in the collagen superfamily lends itself to the various functions that collagens perform within the body. Primarily, they play an important structural role in maintaining the physical integrity and mechanical strength of various tissues such as bone, skin, and muscle [12]. Mutations in the genes encoding for collagen proteins can lead to severe diseases such as osteogenesis imperfecta, osteoporosis, Ehlers–Danlos syndrome and epidermolysis bullosa, to name a few [12]. It is important to recognize that collagen is not merely a structural element; collagens are also responsible for conveying critical biochemical cues to cells. At a cellular level, functions such as cell adhesion, migration, metabolism, and proliferation are all regulated by cues provided by collagen [13,14,15]. Changes in the type of collagen expressed in a tissue can be used as a hallmark to identify various pathologies [16,17]. 

The ubiquitous nature of collagen, along with its strong mechanical properties, has resulted in its use as a biomaterial for various applications, dating back several decades [18,19]. Even with the advent of many types of synthetic polymers, the use of collagen I as a biomaterial remains widespread, as it is a readily sourced and easily fabricated scaffold that intrinsically presents physiologically relevant cues to modulate cell behavior [20]. As researchers have pursued the development of platforms that possess both biomimetic features and a high degree of controlled tunability, the use of collagen as a scaffold biomaterial has expanded from pure collagen gels to engineered collagen-mimetic environments. In this review, we aim to provide a novel contribution to the literature by applying a holistic view of the broad field of engineered collagen matrices, discussing a wide variety of approaches that have been taken in pursuit of such collagen-mimicking materials.

## 2. Pure Collagen I Matrices

There has always been a need for materials to repair the human body, and naturally-occurring materials, including collagen, have been widely explored for this purpose [21]. For reasons of availability, such materials are commonly derived from xenogeneic or allogeneic sources. Scaffolds derived from pure, unmodified collagen I were among the first materials to be used to generate tissue-engineered structures [18]. Natural collagen can be sourced from numerous species; collagen derived from bovine, porcine, rodent, human, and marine sources is commercially available [22,23]. Collagen sourced from different animals does exhibit differences in primary amino acid sequence, as well as in properties such as immunogenicity and potential for disease transmission. For example, mammalian collagens (e.g., from bovine, porcine and rat sources) can induce a significant inflammatory response in humans [24]. In fact, it is estimated that 3% of the population is allergic to bovine collagen [25]. Despite this disadvantage, type I collagen from bovine and porcine sources is found in a wide variety of clinically used materials due to a long, successful history of use [26,27]. Marine collagen is considered to be significantly less immunogenic and is recognized by the FDA as being ‘safe’ [24]. Although some differences have been noted in the physical properties of collagen scaffolds derived from different animals [28], many physical characteristics of collagen I scaffolds (e.g., stiffness, swelling) have not significantly varied with source [29].

The use of human collagen could eliminate some concerns associated with xenogeneic sources. However, while human collagen is commercially available, it is not economically feasible to purchase in large quantities, thereby motivating the development recombinant human Type I collagen (rhCollagen). One method of rhCollagen production is by genetically engineering tobacco plants to include five genes that cause these plants to express rhCollagen [30]. rhCollagen fabrication has also been proven in bioreactor-based eukaryotic production systems, in both mammalian and insect cell culture, and in several transgenic production systems [31,32,33,34,35]. While rhCollagen represents a promising advancement in collagen sourcing, the production of functional collagen is highly dependent upon a large number of post-translational modifications, which can be difficult to achieve in recombinant systems. Specifically, co-expression of prolyl-4-hydroxylase (P4H) remains a challenge in these systems; P4H is responsible for hydroxylation of prolines, which is essential for fibril formation and resistance to proteolytic degradation [36,37].

**Figure 1:**
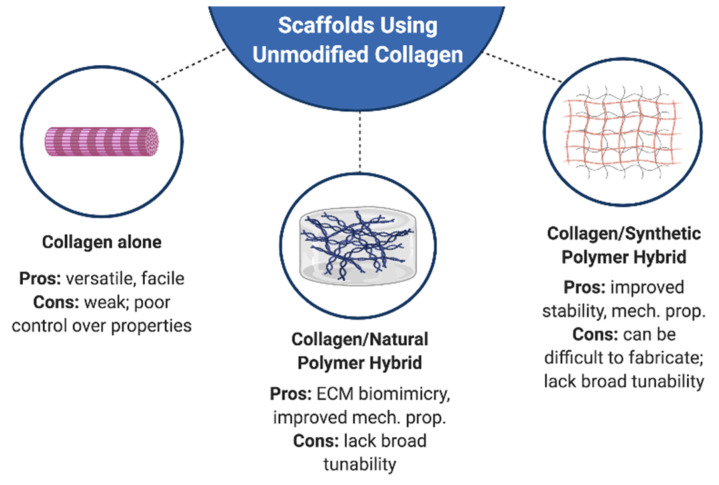
Overview of common approaches to make tailored scaffolds using unmodified collagen I. Figure created using BioRender.com.

In the context of tissue engineering, scaffolds comprised of native, full-length collagen offer numerous attractive features. Advancements in fabrication techniques have permitted the creation of a variety of collagen scaffold forms, including sponges, hydrogels, films, fibers, and meshes (Figure 1). Depending upon the fabrication method, cells can be readily encapsulated within these matrices. These collagen scaffolds can provide to cells a wide range of biochemical and biophysical cues that are intrinsic to the structure of native, full-length collagen. Specifically, they contain numerous adhesive peptide sequences that interact with integrin receptors, they contain peptide motifs that are targeted by matrix metalloproteinases (MMPs) to degrade the scaffold in a cell-responsive manner, and they present fibers that provide topographical and contact guidance cues to cells [38]. In many cases, such scaffolds have been used as the ‘gold standard’ for providing a simplified in vivo-like environment to cells, and they can serve as a positive control against which other collagen-mimetic materials are compared. However, as the triple helical structure of collagen is easily disrupted by environmental conditions (e.g., pH, temperature), the types of engineering manipulations that can be achieved using pure collagen are limited (Figure 1). 

### 2.1. Types of Collagen Matrices

Collagen may be formed into porous, three-dimensional sponges, where it has been used as wound dressings, tampons, 3D culture of various cell types, regenerative endodontic therapy, and as bone-cartilage substitutes [39,40,41]. Commercially available sponges made from collagen I include products such as SpongeCol^®^ (Advanced Biomatrix, Carlsbad, CA, USA), Helistat^®^ (Integra Life Sciences, Princeton, NJ, USA) and SkinTemp II^®^ (Human Biosciences, Inc., Gaithersburg, MD, USA), and are used clinically for applications such as hemostasis and dermal wound healing. There are several methods of manufacturing a collagen sponge; the most common involves creating a slurry of pure collagen, which can then be formed into a sponge by way of lyophilization, free salt leaching, or solvent casting [42], of which lyophilization tends to be the most common. The resulting structure is then commonly crosslinked using an agent like formaldehyde to improve its stability, although it is important to note that the aldehyde groups can exhibit toxicity towards cells [39,43]. Although classic sponges still remain in use, advancements in this area have enabled the creation of multi-modal sponge materials that also serve a drug delivery function. Collagen sponges have been employed by many groups as a delivery vehicle for antibiotics [44,45] and growth factors that stimulate bone formation, such as bone morphogenetic protein-2 [46,47]. The existing use of collagen sponges for clinical applications and their ease of loading with drug/biomolecules makes them an attractive drug delivery carrier. However, unlike other components of the ECM, collagen I does not exhibit a strong ability to sequester biomolecules, resulting in burst release of incorporated agents, which can produce inflammation and other deleterious effects [48]. 

Collagen I hydrogels are highly versatile, as their physical properties and fibrillar architecture can be controlled via factors such as pH, ionic strength, temperature, and collagen concentration. Collagen hydrogels have been used across virtually all types of tissue engineering research, cosmetic applications due to ease of injectability, drug delivery, intervertebral repair and wound healing [49,50,51,52]. The fabrication of collagen gels most frequently utilizes type I collagen that is solubilized using pepsin digestion, acid-salt precipitation, acid extraction, or collagenase. The solubilized form can then be polymerized by customizing the factors mentioned earlier to form gels [53]. The phenomenon behind collagen polymerization involves a nucleation stage, followed by a rapid-growth stage. At a neutral pH and a physiological temperature of 37 °C, the resulting collagen gels tend to have smaller pore sizes along with a mesh-like network of thin collagen fibrils with minimal bundling [54]. Lower pH and lower temperature both lengthen the nucleation phase, thereby causing the formation of longer collagen fibers with larger pores and increased thickness [55,56]. Collagen gels readily allow encapsulation of cells and represent a facile way to provide cells with a biomimetic ECM environment, and have thus been widely employed across thousands of in vitro investigations [53,57]. However, this scaffold platform presents significant limitations with respect to achieving a wide range of mechanical properties or tuning the scaffold characteristics.

Fibrous mesh scaffolds composed of collagen can be formed using electrospinning methods. Due to the high surface area to volume ratio and porosity, collagen fibers are excellent for wound healing, drug and gene delivery, and tissue engineering skin [58,59]. Electrospinning of collagen solutions can result in a mesh-like pattern or highly aligned fibers, producing fibers at a nanometer scale that allows the matrix to mimic the structural and functional profile of in vivo collagen fibers [60]. Electrospinning techniques have been applied to yield collagen fibers with a characteristic 67 nm banding pattern, which is commonly found in native collagen [61]. A variant of electrospinning to form collagen fibers is direct-write electrospinning, which is a form of 3D printing. One group was successful in using this method to ‘print’ collagen into a highly ordered 3D scaffold [62]. 

Finally, a newer approach to forming matrices from pure collagen is 3D bioprinting [63]. Bioprinting offers a level of geometric and architectural flexibility that cannot be achieved with traditional scaffold formation methods. Using pure collagen as a bioink, researchers have made structures such as in vitro skin substitutes for studying wound healing [64], as well as heart valve scaffolds that were implanted subcutaneously in rats [65]. However, because typical formulations of pure collagen exhibit poor mechanical properties, it remains fairly uncommon to use pure collagen in bioprinting [63]. The feasibility of bioprinting pure collagen can be improved by using high-concentration collagen solutions [66], although these concentrations may be above what would be found in native tissue. In general, collagen is combined with either a temporary, supporting matrix structure, or blended directly with another polymer in order to perform bioprinting. 

Although employed in different applications, these different types of scaffolds fabricated from pure collagen share some common drawbacks. Collagen has a tendency to degrade rapidly, causing failure of the matrix due to weakened mechanical properties [58,67]. Furthermore, even prior to degradation, pure collagen scaffolds possess poor mechanical properties, limiting their use as scaffolds where high mechanical strength is required [68]. Although crosslinking agents may be employed to improve scaffold stiffness [69,70], these approaches have also been associated with limitations with respect to compatibility with cell encapsulation and relatively modest impacts on scaffold properties. Moreover, the ability to precisely tune collagen scaffold properties has remained a challenge. The properties of pure collagen scaffolds are most often altered by changing the collagen concentration or adjusting parameters of the fabrication technique, but these variables offer limited range over precisely controlling scaffold characteristics. 

### 2.2. Cross-Linking of Collagen Matrices

Owing to the methods used to extract and purify collagen, the resulting scaffold product tends to have poorer mechanical properties compared to native collagen I. To combat the lack of mechanical strength, low structural stability, and poor enzyme resistance in pure collagen-based scaffolds, there are several physical and chemical treatments available that can cross-link collagen on an intermolecular level. In the native, cellular microenvironment, collagen may be crosslinked by enzymes such as lysyl oxidase and transglutaminase [70,71]; in vitro, such crosslinking enzymes may be added to achieve modest increases in collagen gel stiffness [72]. Another natural method of crosslinking collagen is the administration of glycating agents (e.g., high concentrations of ribose), which yields the formation of advanced glycation end-products and stiffening of collagen [73]. Fixatives such as glutaraldehyde have long been used to crosslink collagen matrices, although this approach is accompanied by numerous limitations, such as problems with carcinogenicity, cytotoxicity, and premature calcification of the resulting scaffold [74,75,76]. Alternatives to glutaraldehyde include reactants such 1-ethyl-3-(3-dimethylaminopropyl) carbodiimide (EDC) and N-hydroxysuccinimide (NHS), which are much less toxic to cells [77]. In recent years, the plant-derived compound genipin has been gaining traction as a collagen crosslinking agent, as it is naturally derived, fast-acting, and has low toxicity [78]. The polyphenolic compound pentalloyl glucose also shows significant promise in stabilizing collagen and improving its resistance to degradation [79].

Finally, collagen itself may be chemically modified with crosslinker molecules that react with each other [80]. Specifically, methacrylate groups can be introduced into collagen by reaction with methacrylic anhydride to yield a photocrosslinkable material. Although the triple helical form of collagen was retained following modification with methacrylate groups, this process did result in a significant decrease in the collagen denaturation temperature, to 36 °C [81]. Scaffolds can be made by employing the usual collagen self-assembly step, followed by exposure to the designated wavelength of light in the presence of a photoinitiator, to catalyze free radical chain polymerization. The resulting scaffolds exhibit significantly increased mechanical properties compared to unmodified collagen. 

## 3. Engineering Hybrid Collagen I Matrices

As noted above, scaffolds fabricated from pure collagen I possess many limitations with respect to their tailorability. Furthermore, the desire for improved mimicry of the in vivo environment motivates the inclusion of additional ECM components in tissue-engineered scaffolds. Fabrication of hybrid collagen matrices, wherein collagen is blended with other components, can enable improved tuning of scaffold composition and physical properties. Because these scaffolds also present native, full-length collagen to cells, they offer similar advantages with respect to tissue engineering as described in Section 2—with the exception that blending with another native ECM component can even further enhance their biochemical mimicry compared to collagen alone. There are several methods to create hybrid collagen matrices, including blending with natural polymers, synthetic polymers, and inorganic materials (Figure 1). The approaches described in this section all refer to collagen I that has not been covalently modified prior to blending with other materials (i.e., ‘pure’ collagen).

### 3.1. Natural Polymers

Most polymers found in nature have the inherent advantage of already containing ligands that are recognized by cellular receptors; biological recognition of these ligands can facilitate processes such as wound healing, cell proliferation, and migration [82]. As discussed in more detail in Section 4.3, collagen itself also contains numerous bioactive peptide sequences [83]. Blending of collagen with other naturally-derived materials allows one to not only merge different types of biological cues, but also different mechanical and architectural features. This approach can yield a more complex, and perhaps more physiologically relevant, matrix environment. Drawing upon the ease of chemically modifying other types of natural materials, these hybrid collagen matrices can also have more tunable mechanical characteristics than collagen alone. Such hybrid scaffolds have been pursued for many years, with the most prevalent natural materials being fibrinogen, hyaluronic acid, chitosan, alginate, and cellulose. With the exception of fibrinogen, these aforementioned biomolecules are polysaccharides, which are often easier to use in scaffold synthesis as they are more readily soluble in varied solvents and do not denature into unfolded structures under conditions like high heat, as is the case with proteins [84]. 

Chitosan is a polysaccharide derived from chitin within the shells of crustaceans. Although it is not a part of the human ECM, it is well-tolerated by the human body. Chitosan also possesses anti-microbial properties that make it an attractive biomaterial for tissue engineering [85]. Alone, chitosan has enjoyed popularity for use in a variety of applications such as drug delivery systems and wound healing [86]. Typically, collagen/chitosan scaffolds are constructed using lyophilization of the polymer blend, followed by crosslinking with agents such as glutaraldehyde [87,88,89]. Blending collagen with chitosan generally allows for greater control over the scaffold degradation rate compared to collagen alone, thereby overcoming an important limitation of pure collagen scaffolds [88]. The stability, swelling, and degradation properties of collagen/chitosan scaffolds can be tailored by altering the ratio of components, as well as changing what type of crosslinking agent is applied [89].

Fibrinogen is native to the human body and is a critical component of the blood clotting process. With the assistance of thrombin, fibrinogen polymerizes to form fibrin, which creates a provisional matrix that stimulates and enables wound healing in the body [90]. Fibrinogen is rich in many bioactive adhesion sites, such as RGD (Arg-Gly-Asp), and has an affinity for factors such as vascular endothelial growth factor. These properties make fibrinogen an attractive biopolymer for scaffolds, especially in combination with collagen, whose own biorecognition sites are sometimes altered during chemical crosslinking. Because fibrinogen and collagen can both self-assemble/polymerize in aqueous solution, they are most frequently combined to make scaffolds in a hydrogel form. For example, collagen and fibrinogen can be mixed with a suspension of cardiomyocytes, which is cast into a PDMS mold before being crosslinked by thrombin to form a collagen/fibrin hydrogel [91]. Fibrinogen can also be combined with collagen using lyophilization as previously mentioned in the context of chitosan [92,93], and can be combined with collagen in the context of 3D bioprinting [94].

Hyaluronic acid (HA) is another component found within the ECM of the human body, primarily within connective tissue. It is an unsulfated glycosaminoglycan (GAG), typically occurring in healthy tissues as a large molecular weight molecule (>1 M Da). HA is recognized by cell surface receptors (e.g., CD44), and contributes to tissue compressive strength, hydration, and lubricity [95]. In line with these properties, cartilage tissue engineering is one of the applications targeted for use of collagen-HA scaffolds, due to the resemblance of collagen-HA scaffolds to the native tissue. Combining HA with collagen is frequently done using the previously mentioned freeze-drying method, followed by use of carbodiimide chemistry (e.g., EDC) to crosslink the scaffold [96,97]. The combination of collagen with HA is relevant to the ECM composition of many tissues, and these scaffolds have been employed to mimic environments such as adipose tissue and aortic heart valves [96,97]. Modulation of scaffold properties can be achieved by altering the concentration of the starting components and the concentration of the EDC [97]. Collagen-HA scaffolds can also be modified to perform drug delivery functions, for example delivery of prednisolone to aid in cartilage repair in osteoarthritis [98]. 

Alginate is a natural polysaccharide originating from brown seaweed and certain types of bacteria [99]. It is highly amenable to chemical modification and exhibits good tunability with respect to physical properties. It has been used in an assortment of hybrid scaffolds for drug delivery, along with bone and skin tissue engineering [100]. A hybrid scaffold of collagen I with alginate is FDA-approved for clinical usage as a dermal wound healing material (FIBRACOL Plus™, Acelity, St. Paul, MN, USA). Alginate is typically merged with collagen to make hydrogels, using calcium chloride as a crosslinker for the alginate, and allowing self-assembly of the collagen [101]. Materials fabricated using this approach have been found to support the function of a variety of cell types primarily in the context of dermal wound healing [102]. Collagen-alginate hybrid materials also perform well in enabling controlled drug release profiles for tissue engineering [103,104,105]. 

### 3.2. Synthetic Polymers

Synthetic polymers do not intrinsically possess bioactive moieties, but their advantages lie in being highly reproducible, with low immunogenicity and easy customization for applications as necessary [82]. Collagen has previously been combined with myriad synthetic polymers such as poly (ethylene glycol) (PEG), poly (ε-caprolactone) (PCL), poly-L-lactide (PLLA), poly (lactide-*co*-glycolide) (PLGA), and poly vinyl alcohol (PVA). 

PLGA is a widely used polymer with a highly controllable degradation rate based on the ratio of lactide to glycolide units, producing lactic acid and glycolic acid as the degradation byproducts. PCL and PLLA are similarly common synthetic biomaterials, and an attractive aspect of all three of these materials is that they are currently used in a broad range of clinical applications [106,107] . PCL, PLGA, and PLLA are frequently fabricated as fibrous mesh scaffolds. Many different approaches have been employed to combine these polymers with collagen, ranging from simple coating of fibers with collagen to electrospinning the blended polymer/collagen solutions. Coating of synthetic polymers with collagen I has demonstrated significantly increased cell proliferation in many applications [108]. Electrospinning to produce PLGA/collagen or PLLA/collagen fibrous scaffolds has produced similar outcomes in applications ranging from vascular to dermal tissue engineering, with the inclusion of collagen significantly increasing cell proliferation compared to the synthetic polymer alone [109,110]. Collagen may also be combined with PLGA or PLLA to form interpenetrating networks (IPNs), where hydrogels or sponges made from unmodified collagen I are used to fill the interstitial spaces within the structure of a PLGA/PLLA fibrous scaffold [111]. This scaffold formation approach allows for the creation of collagen-containing matrices that are stabilized with respect to both mechanical properties and degradation by the surrounding synthetic polymer network.

Blends of PEG with unmodified collagen are generally fabricated in hydrogel form, offering tunable degradation profiles, high water content, and biocompatibility. However, while this seems like it should be a straightforward approach, simple blending of pure collagen with common crosslinkable PEG derivatives (e.g., PEG-acrylate) can often result in phase separation of the components, yielding uneven polymerization and structure [112]. Yet, there are still approaches whereby the two components can be combined, mostly via reaction with primary amine groups on collagen. One novel approach first formed PEG-based microgels using acrylate crosslinking, and these microgels were subsequently modified with NHS groups and reacted with a multi-arm PEG-amine and collagen to form a hydrogel scaffold [113]. This method of scaffold formation permitted collagen concentration to be altered without affecting scaffold stiffness, which is advantageous for creating controlled scaffold environments to probe specific biological questions. In another approach, multi-arm PEG was functionalized with succinimidyl ester groups and combined with collagen to form a hydrogel [114]. In this situation, the succinimidyl ester on PEG can react with primary amine groups in collagen, forming a crosslinked network that may have applications in filling tissue defects or facilitating wound healing [114]. 

### 3.3. Inorganic Composite Materials

A naturally occurring composite consisting of collagen and inorganic material is bone. There are multiple collagen-calcium phosphate materials that are clinically used as bone void filler materials, including Healos^®^ (Depuy, Raynham, MA, USA) and Collagraft^®^ (Zimmer, Warsaw, IN, USA). Such collagen-calcium phosphate materials have been in use for close to 20 years, but development of new technologies in this area continues in order to further improve upon the properties and functionality of these scaffolds [115]. For example, a new technique termed “iterative layering” freeze-drying was implemented to fabricate a matrix comprised of well-distributed HAp within collagen [116]. This scaffold environment presented a highly interconnected porous system capable of promoting osteogenic differentiation [116]. Collagen has also been combined with beta-tricalcium phosphate to form a bioink that can be bioprinted to create scaffolds that supported osteogenic differentiation [117]. Although the rapid degradation of collagen is commonly viewed as a drawback in collagen-based biomaterial systems, it is actually an advantage in the case of collagen-HAp materials, where it helps in achieving degradation on a time scale that is relevant for tissue regeneration. Finally, there are also many ways to combine of all these categories of materials to form matrices; for instance, PLGA-Collagen-HAp composite nanofibers were found to outperform collagen-HAp fibers alone with respect to promoting bone formation [118].

## 4. Engineering Matrices from Collagen-Derived Materials and Sequences

**Figure 2:**
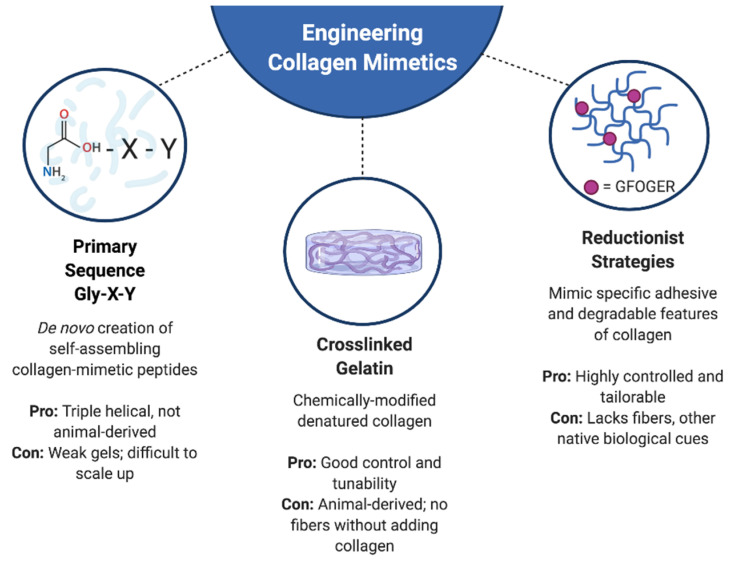
Overview of common approaches used to engineer collagen-mimetic matrices. Figure created using BioRender.com.

Engineered matrices have evolved to use not only pure collagen in scaffold fabrication, but also collagen-derived materials along with various mimics that aim to replicate particular features of collagen for specific purposes. With respect to tissue engineering, many of these scaffolds offer a level of tunability that is not attainable with pure collagen. However, this tunability can come at the expense of full biological functionality. Scaffolds made from collagen-derived materials or sequences do not present the complete collagen molecule in its native configuration, meaning that encapsulated cells will not interact with the full range of biochemical and biophysical cues provided by native, full-length collagen. The omission of such cues can impact the function of encapsulated cells; for example, there are applications where the presence of fibers is necessary in order to support physiological cell behavior [112]. Balancing the need for a tailorable environment with the need for appropriate physiological mimicry is an ongoing goal in the development of new types of collagen-mimetic materials. The content in this section discusses engineering approaches that involve chemical modification of collagen I molecules as well as synthetic recreation of collagen-mimetic moieties and features (Figure 2).

### 4.1. Gelatin-Based Matrices

Gelatin is an irreversibly denatured form of collagen, derived by applying an acidic or alkaline treatment followed by a thermal denaturation of collagen’s structure [119]. After the hydrolytic degradation process, gelatin exists as a linear polymer with a nearly identical amino acid composition as collagen. Thus, gelatin shares many properties with collagen, such as molecular structure, biocompatibility and biodegradation. The benefits of gelatin in comparison to collagen are its lower cost, improved solubility in many solvents, and relative ease of modification. However, there are drawbacks to the use of gelatin in lieu of collagen. The linear structure of gelatin means that many of its peptides are not presented in the correct configuration that is necessary for cellular recognition [120,121]. Moreover, gelatin lacks the ability to form the type of robust fibrillar structures achieved with collagen, which can impact mechanical and degradation properties [122], as well as cellular processes that depend upon topographical or architectural cues [123].

Gelatin is a highly versatile biomaterial and may be used to form scaffolds with or without prior chemical modification, as well as blended to create hybrid scaffolds with other biomaterials. Unmodified gelatin is capable of gelling at low temperatures (e.g., 4 °C), although these gels are unstable at physiological temperatures, and thus require subsequent crosslinking by chemicals such as genipin [124,125]. Physical properties of these gels can be controlled by initial gelatin concentration, as well as variation of temperature during crosslinking [125]. Other hybrid scaffolds that include unmodified gelatin have been made using many of the same polymers noted in Section 3.1 and Section 3.2, such as PLGA and various polysaccharides [122]. For example, PLGA/gelatin scaffolds can be formed via solvent casting, wherein a solution of PLGA and gelatin is left to undergo solvent evaporation on a customized mold [126]. Scaffolds made in this manner can be manipulated via changes in the PLGA/gelatin ratio, and have been used to direct mesenchymal stem cell differentiate into myocardial cells [126]. 

Recent years have seen a significant expansion in the use of methacrylated gelatin (GelMA) as a scaffold material [127]. Gelatin can be readily methacrylated, yielding a photoactive product that be crosslinked using free radical polymerization (typically under UV light) to yield scaffolds with a wide range of mechanical properties [128]. The mechanical properties of GelMA matrices can be controlled by the extent of methacrylate modification, concentration of GelMA in pre-polymer solution, gelation temperature, and gelation time, thereby providing many options for tuning the scaffold features. Scaffolds fabricated solely from GelMA are highly versatile and tailorable, and have found use in a large and diverse range of applications, ranging from bone regeneration to angiogenesis to neural stem cell differentiation; more thorough reviews focused on the topic of gelatin methacrylate provides numerous such examples [127,129].

Moreover, the methacrylate groups in GelMA may be reacted with acrylates, methacrylates, or thiols (via Michael addition or thiol-ene reaction) in other materials to form copolymerized, hybrid structures. For example, ECM components such as HA and chrondoitin sulfate (CS) are readily methacrylated (or acrylated), as are other non-native polymers such as PEG; GelMA can be combined with any of the aforementioned molecules to form copolymerized networks. Scaffolds made from GelMA and a GAG (e.g., HA or CS) can offer more complex biomimicry of a native tissue than GelMA alone. Moreover, by blending different ECM components, one can start to mimic complex biological or pathological events in a controlled manner. For example, GelMA was blended with methacrylated HA and CS to form scaffolds that resembled both healthy and diseased heart valve tissue in order to elucidate the order of events in disease progression [130]. The tailorability of the GelMA platform enabled the incorporation of varied amounts of GAGs while maintaining constant scaffold stiffness, thereby allowing the examination of the role of the ECM alone, without being confounded by other simultaneous changes in variables. 

**Figure 3:**
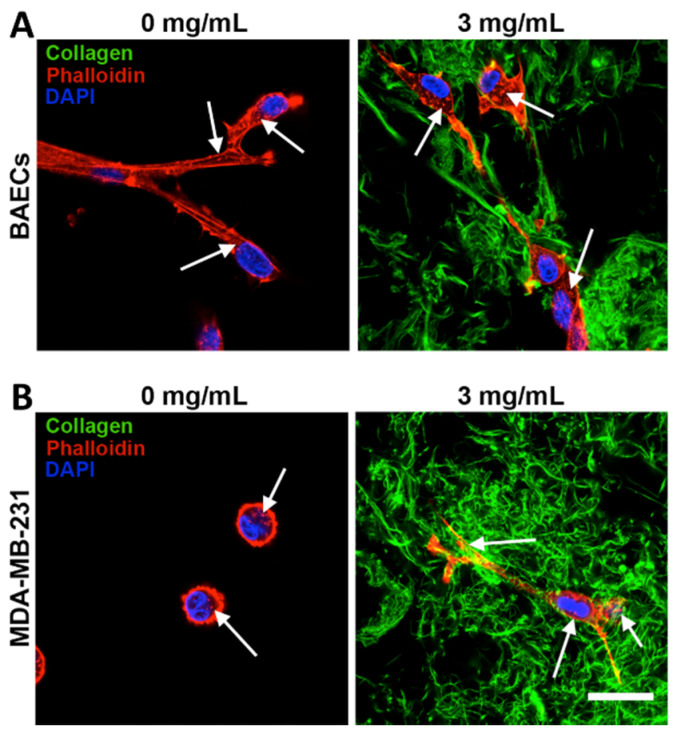
Effect of collagen fibers on single-cell morphology varies with cell type. (**A**) Bovine aortic endothelial cells (BAECs) exhibited a spread morphology in both the absence and presence of fibrillar collagen in 2 kPa GelMA/collagen gels, whereas (**B**) MDA-MB-231 cells remained rounded with no significant protrusions when fibrillar collagen was not present, but elongated and adopted a spread morphology when fibers were added to the 2 kPa scaffold. The inability to spread in the non-fibrillar scaffold prohibited the ability of the MDA-MB-231 cells to invade their surrounding matrix. White arrows point to actin staining puncta. Figure adapted from [112] and reprinted with permission.

As noted above, however, GelMA lacks a critical feature that makes collagen so unique: fibers. While there are many scaffold platforms in widespread use that do not include fibers (e.g., PEG hydrogels), there is mounting evidence that fibers are essential for mimicking certain biological processes (Figure 3) [131]. Fibers are widely recognized as playing a critical role in tumor cell metastasis [132], and fibrotic diseases, by definition, are dependent upon having a fibrillar environment [133]. For many years, it has been a challenge to develop a collagenous material that is easily and broadly tunable in terms of physical properties, while also allowing presentation of a fibrillar architecture. Recently, a type of interpenetrating network was developed to capitalize on the tunability of GelMA, while also incorporating collagen fibers. To make these scaffolds, unmodified collagen I is blended with GelMA, and the former is allowed to undergo fibrillogenesis, while the latter is subsequently crosslinked around the collagen fibers via exposure to UV light [112]. Using this approach, one can independently vary scaffold stiffness, collagen fiber density, and overall protein content, while also allowing for encapsulation of cells [112]. Importantly, application of these scaffolds has both confirmed that fibers are necessary for the procession of certain cellular functions, and has revealed novel biological insights related to tumor cell invasiveness [134]. These materials also open the door for incorporation of additional elements, such as methacrylated GAGs, to increase the complexity of tissue biomimicry.

### 4.2. Collagen Mimics

Numerous synthetic approaches have been applied to develop materials that mimic specific biochemical and physical features of collagen. For example, one approach to generating collagen-mimetic materials is to directly synthesize short, simplified collagen-mimetic peptides (CMPs) consisting of repeating Gly-X-Y units in order to produce triple helical chains. Techniques such as solid phase peptide synthesis and native chemical ligation can be applied to construct these CMPs directly from amino acid building blocks [135]. The sequence of CMPs can be tailored to improve its triple helical structure and self-assembly into fibrils [136]. Many groups have employed this approach to generate self-assembling CMPs, although there has been mixed success in being able to form robust fibers and gels based upon these molecules [137,138,139,140]. While CMPs may not always be amenable to forming stand-alone gels, the individual peptides have still shown utility when incorporated into other scaffold materials. For example, inclusion of CMPs in a PEG-based hydrogel system resulted in improved retention of cell-secreted collagen within the scaffold, presumably due to the secreted collagen being able to interact with the CMP; CMPs are known to associate with collagen via a strand invasion process [141]. In theory, CMP-based materials offer the advantage of providing controlled environments and avoiding xenogeneic sources of collagen. As they are able to assemble into higher order structures, they can also be manipulated to assemble at specific triggers. However, these methods can be expensive in terms of time and money when natural collagen is readily available to source.

Finally, another method of engineering collagen-like structures is to simulate only the morphological structure of collagen without needing the requisite amino acids for collagen. CMPs contain amino acids that follow the Gly-X-Y formulation of collagen. However, it is possible to manufacture self-assembling fibers that mimic the architecture of native collagen without the Gly-X-Y sequence. For example, methacrylated gellan gum (MeGG) was combined with a countercharged chitosan (CHT) in a polydimethylsiloxane (PDMS) channel to assemble aligned fibers that resembled the dark/light pattern reminiscent of collagen D-periodic bands [142]. Others have fabricated dextran fibers with similar dimensions as native collagen fibers [143] and found that fibrillar topography may be more influential than biochemical identity (e.g., having an actual collagen peptide sequence) in guiding cellular responses [131]. Such methods of simulating collagen are attractive as they do not require the production of expensive peptides and can be developed using natural polysaccharides. Moreover, these techniques allow researchers to separately probe the roles of peptide identity and fibrillar architecture.

### 4.3. Reductionist Approaches to Engineering Collagen-Mimetic Matrices

A final approach to engineering collagen-mimetic environments is to reduce collagen to its minimum bioactive elements. This approach concentrates on incorporating specific, bioactive, collagen-derived peptide sequences into other scaffold materials. Like most adhesive ECM proteins, collagen contains the RGD (Arg-Gly-Asp) peptide motif [83]. However, the RGD present in triple helical collagen sequences is not available for recognition by integrins, meaning that cell adhesion to fibrillar collagens is typically RGD-independent [83]. Rather, the peptide sequences DGEA (Asp-Gly-Glu-Ala) and GFOGER (Gly-Phe-OHPro-Gly-Glu-Arg) are the dominant adhesive moieties in collagen I. Integrin recognition of the GFOGER sequence is dependent upon it being in a triple helical conformation. By appending a cysteine to either of these peptides, one can attach them to polymers with thiol-reactive groups (e.g., methacrylate, acrylate, maleimide) [144,145]; alternatively, one can use carbodiimide chemistry to react the peptides with an amine- or carboxyl- containing polymer. These approaches ultimately yield a scaffold environment that displays collagen-specific bioactivity. Adhesive peptides are generally incorporated into a non-adhesive background material (e.g., alginate, PEG), so that one can specifically evaluate the role of the peptide modification, in the absence of factors that might promote non-specific cell adhesion. For example, DGEA was bound to alginate using carbodiimide chemistry and found to enhance osteogenic differentiation of mesenchymal stem cells [146]. Incorporation of GFOGER into scaffolds made from multi-arm PEG-maleimide has similarly shown that it can improve osteogenic outcomes, including the enhancement of bone repair in vivo [147]. The application of GFOGER-modified materials is not limited to bone, as such materials have also been found to support the culture and function of intestinal enteroids and endometrial spheroids [148] or mimic the tumor microenvironment [149], amongst other applications.

Reductionist approaches may also be used to mimic the degradation characteristics of collagen. In this scenario, a minimum peptide sequence needed for matrix metalloproteinase (MMP) recognition is identified and incorporated into a scaffold. Similar to adhesive sequences, these MMP-degradable sequences are commonly incorporated within a PEG scaffold background; typically, the peptide is modified with a cysteine on both ends, and then serves to join together thiol-reactive PEG chains. Many sites within the collagen molecule can be targeted for cleavage by various MMPs [150], with the peptide sequence GPQGIAGQ (sometimes abbreviated as ‘GIA’) being included in several biomaterial systems [151,152]. Because biomimicry of the native ECM requires the ability to support both adhesion and degradation, incorporation of these MMP-degradable sequences is usually performed in addition to the adhesive modifications noted in the previous section. The degradation profile of MMP-sensitive materials can be further modulated by tailoring the MMP target sequence. For example, a simple amino acid substitution (from A to W) in GPQGIAGQ can significantly improve its sensitivity to MMP-1 [153], while different MMP target sequences offer different levels of specificity to individual MMPs [153]. Put together, these reductionist approaches can yield highly controlled and characterized scaffold environments that mimic key aspects of collagen bioactivity. 

## 5. Opportunities

Almost 35 years after its use as a scaffold in one of the first instances of modern tissue engineering [18], collagen remains a widely used biomaterial. However, those intervening decades have been accompanied by innovative developments in the creation of collagen-based scaffolds, ranging from blending with other materials to de novo synthesis of collagen-like peptides. Yet, there remain many opportunities to further engineer and optimize collagen-mimetic scaffolds.

A rapidly developing area in the fabrication of engineered collagen matrices focuses upon replicating the complex geometrical and architectural features of collagen. While the majority of synthetic collagen-mimetic approaches have focused on its biochemical attributes, there is mounting evidence supporting the importance of collagen architecture in guiding cell function [112,134,143,154]. Many elements of the nano- and micro- structure of collagen are important for guiding proper cell responses, as well as for providing desired mechanical characteristics. For example, traditional collagen gelation techniques do not yield fiber bundles that are physiological in size. Although recent advancements have yielded scaffold conditions where large fiber bundles (up to 350 μm) can be produced by encapsulated cells [155], there is also a desire to be able to create scaffolds where those fiber dimensions are physiological from the outset so that one can probe biological and pathological questions related to fiber diameter and bundling. Fiber size is also important for improving the mechanical strength of collagen scaffolds to better mimic the in vivo environment [155].

The creation of collagen scaffolds that better replicate the fiber arrangement/alignment seen in both healthy and pathological tissues is also an emerging area of emphasis and innovation. Typical collagen-based scaffolds present isotropic fiber organization, in contrast to the anisotropic organization seen in vivo. Again, this architectural feature is important for eliciting physiological cell behaviors; for example, fiber anisotropy is required to align cells such as cardiomyocytes, and fiber alignment perpendicular to solid tumors is thought to be a critical component of tumor metastasis [132]. Approaches to yield aligned collagen fiber scaffolds include the use of microfluidic platforms [156] and the application of anisotropic strain during collagen gelation [157]. Techniques such as multi-photon excited fabrication take the architectural replication a step further and allow for direct recapitulation of exact, tissue-specific fiber features based upon scans of native tissue [154]. However, some of these platforms do not permit cell encapsulation at the time of scaffold fabrication, and have thus been limited to studying these fibrillar interactions in 2D [156]. Of the platforms that permit concurrent cell encapsulation, many utilize specialized techniques that are relatively low-throughput and would not be readily accessible to many researchers. Others have demonstrated that methods to control fiber alignment concurrently change multiple other physical properties of the scaffold [158], which can introduce problems with data interpretation, as numerous variables are changing at once. Thus, to advance our ability to study and recapitulate conditions where fiber organization plays a critical role in tissue function, there remains a need for simplified techniques for producing collagen-based scaffolds with controlled fiber organization. There is also a need to blend the ability to control scaffold composition and properties with the precise presentation of fibrillar architecture.

Innovations in the next generation of engineered collagen scaffolds can also include further combinations with other complex ECM structures, including other types of collagen, as well as expanded options for non-toxic crosslinking treatments. This review has focused exclusively on collagen I, but many other types of collagen are present in tissues, often in abundance. The options for engineering other types of collagen in a scaffold context are limited, and mostly focus on reductionist approaches or their use as coatings. For example, type IV collagen has been used to coat polyacrylamide gels to model defects in the basement membrane, and type IV collagen peptide motifs responsible for cell adhesion, spreading and motility have been identified [159,160]. More options exist for engineering type II collagen scaffolds; there are mammalian and insect-based expression systems for the production of recombinant type II collagen, which has potential as a material for cartilage tissue engineering [161,162,163]. However, overall, type II collagen remains largely unexplored as a material for engineered matrices. Finally, the continuing need for non-toxic methods to crosslink collagen-based matrices is often overshadowed by the flashier developments in scaffold fabrication and biomaterials chemistry, but is no less important. The crosslinker toolbox available for researchers to use on cell-containing scaffolds remains fairly limited [164], but developments in this area could greatly improve our options with respect to fabricating scaffolds and controlling their properties.
